# Work Resumption Rate and Migrant Workers' Income During the COVID-19 Pandemic

**DOI:** 10.3389/fpubh.2021.678934

**Published:** 2021-05-21

**Authors:** Jiaxiang Li, Baoju Chu, Nana Chai, Bi Wu, Baofeng Shi, Feiya Ou

**Affiliations:** ^1^College of Economics and Management, Northwest A&F University, Yangling, China; ^2^State Grid Fujian Electric Power Co. Ltd., Fujian, China; ^3^School of Economics, University of Nottingham, Ningbo, China; ^4^Research Center for Rural Economy, Ministry of Agriculture and Rural Affairs, Beijing, China; ^5^Business School, University of Manchester, Manchester, United Kingdom

**Keywords:** COVID-19, migrant worker, work resumption rate, income, scenario analysis

## Abstract

The COVID-19 public health crisis has quickly led to an economic crisis, impacting many people and businesses in the world. This study examines how the pandemic affects workforces and workers' income. We quantify the impact of staggered resumption of work, after the coronavirus lockdowns, on the migrant workers' income. Using data on population movements of 366 Chinese cities at the daily level from the Baidu Maps-Migration Big Data Platform and historical data on the average monthly income of migrant workers, we find that the average work resumption rate (WRR) during the period of the Chinese Lantern Festival was 25.25%, which was only 30.67% of that in the same matched lunar calendar period in 2019. We then apply Gray Model First Order One Variable [GM (1, 1)] to predict the monthly income of migrant workers during the period of the COVID-19 pandemic. We show that, if without the influence of the COVID-19 pandemic, the average monthly income of migrant workers in 2020 will be expected to increase by 12% compared with 2019. We further conduct scenario analysis and show that the average monthly income of migrant workers in 2020 under the conservative scenario (COS), medium scenario (MES), and worse scenario (WOS) will be predicted to decrease by 2, 21, and 44%, respectively. Through testing, our prediction error is <5%. Our findings will help policymakers to decide when and how they implement a plan to ease the coronavirus lockdown and related financial support policies.

## Introduction

In December 2019, a novel coronavirus disease (COVID-19 for short) appeared in Wuhan, China (1, 2). Then, in early 2020, COVID-19 followed in other parts of the world. As of March 22, 2021, there are approximately 123.87 million confirmed cases of COVID-19 in nearly 200 countries and about 2.73 million people have lost their lives. The effective prevention and control measures of this epidemic should become the top priority of all governments around the world. The Chinese government activated first-level emergency response in order to deal with the outbreak of this epidemic. A series of unprecedented measures, such as city blockade, suspending public transport, imposing gathering and movement restrictions, extended school closures, and factory suspensions, has been implemented rapidly to contain and delay the spread of the COVID-19 (3, 4). China has managed to contain the virus through the use of those draconian control measures, but at a heavy price. The economic fallout from this pandemic is threatening China and global growth and financial stability. Business activity has ground to a halt in most sectors such as aviation, travel, and tourism. Unemployment soars significantly. After months of lockdown in China, the government is slowly easing emergency measures. Industries are allowed to reopen and people are returning to work. But, officials and analysts remain concerned about the negative outlook for China's economic growth in 2020 and in particular about the strain a downturn would have on rural areas and low-income regions. Therefore, it is very important to assess the impact of the COVID-19 on the Chinese labor market and low-income groups, such as migrant workers who most likely lost their jobs during this crisis, in order to provide empirically based implications for government policies that aim to orderly organize workers to resume work and production and guarantee farmers' income after the epidemic alleviates.

According to the National Bureau of Statistics, migrant workers make up about one-third of China's vast workforce. The number of migrant workers reached nearly 291 million in 2019, with 60% of them employed outside the countryside and in cities that may be far away from their official home base (5). Affected by the COVID-19, the Chinese government has taken measures to restrict population mobility (3, 4), and many industries have also been forced to shut down and postpone resumption of work. The outbreak of COVID-19 and the control measures of the Chinese government have put forward higher requirements for the survivability of small- and medium-sized enterprises (SMEs), which are the main carriers of migrant workers. If SMEs go bankrupt, a large number of migrant workers will face the risk of unemployment, thus triggering a social employment crisis and threatening the stability of the entire society.

Moreover, the impact on the income of migrant workers will bring huge pressure to increase farmers' income. In 2019, the per capita disposable income of rural residents in China was $2,322.39. Among wage income, operating income, net property income, and net transfer income, the wage income accounts for the largest proportion, which is 41% (5). Therefore, it is not difficult to infer that the impact of the epidemic on migrant workers will directly affect the farmers' annual income. Secondly, as the main population that flows during the Chinese Spring Festival, migrant workers are affected by factors such as economic conditions, social security, ability of information access, community support, etc., so they are at high risk and highly vulnerable during the epidemic. Compared with public institution staff, they almost have no social security. Once the risk comes, it's even worse. We can foresee that if tens of thousands of migrant workers return to work without any hope, it will not only cause large-scale farmers returning to poverty but also lead to losing a strong support for building a moderately prosperous society in all respects. The consequences could be disastrous.

In China, the employment and income issues of nearly 300 million migrant workers have received considerable attention from government and researchers. China has witnessed deepening reform and a continuous, large-scale exodus of rural labor since the Reform of 1978 (6, 7). Rural labor was gradually showing a trend toward the private sector (8) and trans-regional and urban areas (9, 10). While the labor force was transferred from the countryside to the city, the employment industry of the rural labor force has expanded from agriculture to non-agricultural fields. The non-agricultural employment of agricultural population provided hundreds of millions of labor force for China's economic development and promoted rapid development of the economy (11–15).

The regular mobility of migrant workers', especially seasonal mobility, is an important condition to ensure the full employment. If the mobility is restricted, it is difficult to secure their jobs. Some scholars pointed out that when public emergencies broke out, the flow of migrant workers was mainly restricted by objective factors such as the adjustment of government policies and the adjustment of internal employment plans of the enterprise. At the same time, cost-cutting initiatives such as requiring staff to take unpaid leave, terminating temporary contracts, and stopping all overtime payments were also adopted by enterprises in order to reduce the impact of public emergencies (16, 17). In addition to objective factors such as external policies and internal adjustments of enterprises, the subjective reluctance of farmers to go out is also an important factor affecting work resumption. After the outbreak of public health emergencies, population mobility is restricted, labor input of enterprises is reduced, and both the industry and national economic output will decrease (18–21). Among them, the secondary industry, which is dominated mainly by manufacturing and construction industries (22–24), and the tertiary industry, such as tourism and catering, which absorbs a large amount of rural surplus labors (16, 25–27), have suffered the most obvious impact (28, 29). Furthermore, some scholars have focused their attention on the analysis of factors affecting the employment of migrant workers. Huang et al. (30) found that migrant workers who are older and less educated are more likely to be unemployed, and workers in industry and construction are more likely to be unemployed than workers in the service sector. Wang (31) also pointed out that gender differences would not affect the unemployment of migrant workers but would affect their willingness to move again. It is not difficult to find that when public health emergencies such as the COVID-19 become stable, the resumption of work will be affected by the epidemic prevention and control policies issued by the government, the adjustment of the internal employment plan of enterprises, and the social panic caused by the epidemic.

However, farmers' income is also closely related to their level of education and skills. Wong et al. (32) pointed out that job mobility among migrant workers is very low since the majority of migrant workers are uneducated and do not have special skills. From the perspective of consumption structure, due to the low-income level of migrant workers, the household consumption structure has been dominated by subsistence consumption, household subsidies, and improvement of living conditions (33, 34). Moreover, due to the unique household registration system of China, even if migrant workers have worked in the city for many years, they cannot enjoy the same treatment as urban residents (35, 36), and they may even suffer employment discrimination (32, 37, 38). Compared with the great contribution of migrant workers to urban economic development, the public services and welfare provided by the urban sector to them are very limited. Even if the Chinese government has gradually relaxed the Hukou regulation and instituted a variety of reforms to the household registration system (39), the social, political, and economic disadvantage of migrant workers will not be changed in a short term due to the size of cities and their skills (22, 31, 40). Thus, the risk tolerance of migrant workers is generally weak (33, 40), and it is difficult to withstand the impact of public health emergencies such as COVID-19 with their own strength. Chan (22) also pointed out that migrant workers are extremely vulnerable to external uncertainties (such as the financial crisis), which will result in sudden unemployment and thus impact their income level. Pan et al. (41) believed that COVID-19 has severely affected the famers' wage income and agricultural income, and it may cause about $100 billion in losses.

In conclusion, we can find that the existing research has made some progress in the impact of emergencies such as natural disasters and financial crisis on labor employment and farmers' income. However, there are significant differences between the impact of COVID-19 and the research above. First, earthquake, financial crisis, and other emergencies cannot cause large-scale population mobility restriction. The impact on the employment of labor and the income of migrant workers is shown as local and regional characteristics. Second, most of the existing literature review and summarize the impact of the event on labor employment or farmers' income after the event. There are few papers that, when the incident occurs, based on the development of the epidemic situation and the level of policy response, promptly measure its impact on the return of labor to work and the income of migrant workers.

In order to evaluate the impact of the COVID-19 on the Chinese labor market and the income of migrant workers who are in a highly vulnerable situation, we obtain the migration data of 366 prefecture-level cities and municipalities directly under the central government from the Baidu migration map in 2019 and 2020 by using the web crawler technology. We then calculated the work resumption rate (i.e., WRR) of migrant workers without COVID-19 in 2019 and with COVID-19 in 2020. Furthermore, we evaluated the impact of the COVID-19 on migrant workers. On this basis, according to the emergency response level of the COVID-19, the average monthly income of migrant workers under conservative, medium, and worse scenarios has been predicted by using the scenario analysis method. We also compared the forecast results with the actual income data of migrant workers in order to test the robustness. Finally, this paper provided countermeasures and suggestions for the labor force to return to work after the epidemic alleviates. Our result shows that the migrant workers' income will increase by 12% compared with 2019 if there is no outbreak of the COVID-19 pandemic. The average monthly income of migrant workers will be slightly affected by the epidemic under the conservative scenario (COS) and it is predicted to decrease by 2%. Then, as time passed, the impact of COVID-19 on migrant workers' income will gradually increase if migrant workers still cannot return to work. Also, their average monthly income is predicted to decrease by 21 and 44% under the medium scenario (MES) and worse scenario (WOS), respectively. The contributions of this paper are as follows: First, we successfully predicted the migrant workers' income in 2020, which are not affected by the COVID-19 with a small sample size by using the Gray Model First Order One Variable [GM (1, 1)] method. Second, we realized the prediction of the impact of restrictions on population flow due to emergencies on migrant workers' income. Third, our model can effectively predict the impact of COVID-19 on migrant workers' income by comparing with the official data published by the National Bureau of Statistics, and it can be used for reference by other countries.

The structure of this paper is organized as follows: The Methodology section introduces the methodology. The Empirical Analysis and Discussions section gives the empirical analysis. The Conclusion and Policy Implications section is the conclusion and policy implications.

## Methodology

In this section, this paper introduced the modeling process of COVID-19 impact on return to work of China's rural labor and migrant workers' income. Firstly, this paper selected the two time points of the Chinese Lunar New Year and the Lantern Festival to calculate the WRR according to the Baidu migration daily data. Secondly, according to the historical data, this paper predicted the average monthly income of migrant workers in 2020 under the inertial scenario (INS) (without the impact of the COVID-19) by using the GM (1, 1) method. Finally, by using the scenario analysis method, this paper predicted the average monthly income of migrant workers in three scenarios: conservative scenario (COS), medium scenario (MES), and worse scenario (WOS).

### The Impact of the COVID-19 on the Return to Work of China's Rural Labor

According to the traditional customs of China, people need to take a bus from the work site to their hometown and get together with their families for the Spring Festival. After that, they can return to work, and most schools begin classes after the Lantern Festival (the 15th day of the first month of the lunar year). Therefore, this paper can select the Spring Festival and the Lantern Festival as two dividing points. By using data from the Baidu migration map, the city's daily population immigration and emigration data can be obtained (42). Then, we can obtain the WRR for each city. Since the outbreak of the COVID-19 occurred in late January 2020, and January 25 is the Spring Festival, we can use the data of a city's moving-out population before the Spring Festival as the data of labor returning home and the data of the city's moving-in population during the Spring Festival to the Lantern Festival as the data of returning workers. The ratio of returning to work in this city during the period of Chunyun^1^ can be approximated.

Let $\underset{t_{1}\to t_{2}}{r_{in}}=\sum_{i=1}^{m}\underset{t_{1}\to t_{2}}{r_{i,in}}$ be the sum of population immigration indexes of all cities in a certain region in the period from *t*_1_ to *t*_2_. $\underset{{t^{\prime }}_{\;1}\to {t^{\prime }}_{\;2}}{r_{out}}\;=\;\sum_{i=1}^{n}\underset{{t^{\prime }}_{\;1}\to {t^{\prime }}_{\;2}}{r_{i,out}}$ denotes the sum of population emigration indexes of all cities in the period from ${t^{\prime }}_{\;1}$ to ${t^{\prime }}_{\;2\;}$. $\underset{t_{1}\to t_{2}}{\;r_{i,in}}$is the population immigration index of the *i*^*th*^ city in the period from *t*_1_ to *t*_2_. $\underset{{t^{\prime }}_{\;1}\to {t^{\prime }}_{\;2}}{\;r_{i,out}}$ is the population emigration index of the *i*^*th*^ city in the period from ${t^{\prime }}_{\;1}$ to ${t^{\prime }}_{\;2}$. The WRR *r* can be given by

(1) $$r\text{=}\frac{\underset{t_{1}\to t_{2}}{r_{in}}}{\underset{{t^{\prime }}_{\;1}\to {t^{\prime }}_{\;2}}{r_{out}}}=\frac{\sum_{i=1}^{m}\underset{t_{1}\to t_{2}}{r_{i,in}}}{\sum_{i=1}^{n}\underset{{t^{\prime }}_{\;1}\to {t^{\prime }}_{\;2}}{r_{i,out}}}$$

where, Equation (1) is the ratio of the population immigration index *r*_*in*_ in the period from *t*_1_ to *t*_2_ and the population emigration index *r*_*out*_ in the period from ${t^{\prime }}_{\;1}$ to ${t^{\prime }}_{\;2}$. It can be used to calculate the WRR. The WRR of the same city in 2019 and 2020 can be used to compare the impact of COVID-19 on workers returning to work. At the same time, we can also make a horizontal comparison between different cities in the same period to find out the difference of impact of the COVID-19 on different cities' WRR.

It should be pointed out that Equation (1) satisfies two basic assumptions. Assumption 1: the demographic structure has not changed much within the selected period. Assumption 2: the purpose of population mobility is relatively single. At present, both of the two hypotheses are established in China. From January 2020 to March 2020, China's population structure does not change much. In addition, China has to experience the Chunyun during the Spring Festival every year. That is to say, people return to their hometown from the city where they work to celebrate the Chinese New Year and then return to the city after the New Year. Therefore, the purpose of population mobility is relatively single. Especially, after the outbreak of the COVID-19, China has taken very strict measures to restrict population mobility, and unnecessary business trips, family visits, and students' return to school have been temporarily banned before the end of February. Then, the main purpose of large-scale population mobility is to return to work.

### The Prediction of the Impact of the COVID-19 on the Income of Migrant Workers

Firstly, we provided the definitions of migrant workers and the average monthly income of migrant workers. Migrant workers refer to the workers who are employed outside their villages and towns for more than 6 months in the year and those who do non-agricultural work in or outside their villages and towns for more than 6 months (5). The average monthly income of migrant workers refers to the average monthly monetary wage of each migrant worker in a certain period. Then, this paper analyzed the impact of the COVID-19 on the income of migrant workers from two aspects. On the one hand, the history data of the migrant workers' income was used to predict the migrant workers' income in 2020. Because the income of migrant workers is not affected by the COVID-19 under this scenario, this paper called it as the prediction of migrant workers' income under the inertia scenario (INS). On the other hand, according to the impact of the epidemic on migrant workers' working time, we considered three scenarios, namely, conservative scenario (COS), medium scenario (MES), and worse scenario (WOS), and predicted the average monthly income of migrant workers in different scenarios.

#### The Prediction Model of Migrant Workers' Income in INS

In the prediction, we found that the sample size of the average monthly income of migrant workers was small. It is due to the annual data of the average monthly income of migrant workers published by the National Bureau of Statistics since 2009. This is consistent with the property of the GM (1, 1) method that can get the high prediction accuracy for the uncertain system with a small sample and poor information (43, 44). A multistep approach for using GM (1, 1) to predict the average monthly income of migrant workers is now presented.

**Step 1**: the raw data accumulation of monthly average income of migrant workers *X*_*t*_.

Let *X*_0_ (*t*) be the average monthly income of migrant workers in the *t*th year and *t* = 1, 2, …, *n*. Given the original data series of monthly average income of migrant workers *X*_0_ = {*X*_0_ (1), *X*_0_ (2), …, *X*_0_ (*n*)}, the *X*_0_ is accumulated to generate the series *X*_1_.

(2) $$\begin{array}{l} {\text{X}}_{1}=\left\{{\text{X}}_{1}\left(1\right),{\text{X}}_{1}\left(2\right),\ldots ,{\text{X}}_{1}\left(\text{n}\right)\right\} \end{array}$$

Where $X_{1}(t)=\sum_{i=1}^{t}X_{0}(i)$

**Step 2**: establish the data matrix *B* and series *Y*_*n*_

(3) $$B=\left[\begin{array}{l} -0.5(X_{1}(1)+X_{1}(2))\;1\\ \ldots \;\ldots \;\ldots \;\ldots \\ -0.5(X_{1}(n-1)+X_{1}(n))\;1 \end{array}\right]$$

(4) $$Y_{n}=\left[\begin{array}{l} X_{0}(2)\\ ...\\ X_{0}(n) \end{array}\right]$$

**Step 3**: calculate parameters *a* and *u* of the GM (1, 1) model

(5) $$\left[\begin{array}{c} a\\ u \end{array}\right]={\left(B^{T}B\right)}^{-1}B^{T}Y_{n}$$

where, *B*^*T*^ is the transpose matrix of *B* and (*B*^*T*^*B*)^−1^ is the inverse matrix of *B*^*T*^*B* in Equation (5).

**Step 4**: calculate the cumulative generation predicted value ${\hat{X}}_{1}$*(t)* of the average monthly income of migrant workers in the *t*th year. It can be given by

(6) $$\begin{array}{l} {\hat{X}}_{1}\left(t\right)=\left[X_{0}\left(1\right)-\frac{u}{a}\right]e^{-a\left(t-1\right)}+\frac{u}{a} \end{array}$$

In Equation (6), *X*_0_(1) is the first data in the original data series *X*_0_. Substituting *t* = 1, 2, …, *n* into Equation (6), respectively, we can get the cumulative generation predicted value ${\hat{X}}_{1}$(1), ${\hat{X}}_{1}$(2), …, ${\hat{X}}_{1}$*(n)* of the average monthly income of migrant workers.

**Step 5**: the accumulated predicted value ${\hat{X}}_{1}\left(t\right)$ is used to calculate the predicted value ${\hat{X}}_{0}\left(t\right)$ of the average monthly income of migrant workers in the *t*th year. That is

(7) $$\begin{array}{l} {\hat{X}}_{0}\left(t\right)={\hat{X}}_{1}\left(t\right)-{\hat{X}}_{1}\left(t-1\right) \end{array}$$

**Step 6**: calculate the average error δ

By comparing the predicted income ${\hat{X}}_{0}\left(t\right)$ of migrant workers with the real income *X*_0_(*t*), the average error rate $\delta \;=\;\frac{1}{n}\sum_{t\;=\;1}^{n}\frac{\left|{\hat{X}}_{0}(t)\text{-}X_{0}(t)\right|}{X_{0}(t)}$ of the GM (1, 1) model can be calculated. If δ < 5%, we can use Equation (7) to predict the average monthly income of migrant workers in 2020. Otherwise, the model needs to be readjusted.

#### Income Prediction of Migrant Workers Based on Scenario Analysis

Combining the emergency response level of Chinese public health emergencies (special major class-I, major class-II, larger class-III, and general class-IV) with the scenario analysis method (45, 46), we can divide the impact of the epidemic on migrant workers into three scenarios.

##### Conservative Scenario

Affected by restrictions on population mobility caused by the COVID-19, farmers cannot go out to work for 1–2 months under COS. At this time, we suppose *a* is the number of months when farmers cannot go out to work under the COS. Then, the monthly average income *X*_0_(*t*)_COS_ of migrant workers under COS can be given by *X*_0_(*t*)_COS_ = *X*_0_(*t*) × (12 – *a*)/12.

##### Medium Scenario

Affected by restrictions on population mobility caused by the COVID-19, farmers cannot go out to work for 3–4 months under the MES. We suppose *b* is the number of months when farmers cannot go out to work under the MES. Then, the monthly average income of migrant workers under MES can be obtained by *X*_0_(*t*)_MES_ = *X*_0_(*t*) × (12 – *b*)/12.

##### Worse Scenario

Affected by restrictions on population mobility caused by COVID-19, farmers cannot go out to work for at least half a year under the WOS. We suppose *c* is the number of months when farmers cannot go out to work under the WOS. Then, the monthly average income of migrant workers under WOS is *X*_0_(*t*)_WOS_ = *X*_0_(*t*) × (12 – *c*)/12. The predictions of the average monthly income of migrant workers under different scenarios are shown in Table 1.

**Table 1:** The average monthly income of migrant workers under different scenarios.

**Scenario category**	**Definition**	**Prediction of the average monthly income of migrant workers *X*_**0**_ (*t*)**
Scenario 1: conservative scenario (COS)	Affected by the NCP, migrant workers cannot go out to work for 1–2 months, which has a slight impact on farmers' income.	Based on the prediction of the average monthly income *X*_0_(*t*) of migrant workers by the GM (1, 1) method in the INS, the income of migrant workers under the slight impact is as follows: *X*_0_(*t*)_COS_ = *X*_0_(*t*) × (12 – *a*)/12.
Scenario 2: medium scenario (MES)	Affected by the NCP, migrant workers cannot go out to work for 3–4 months, which has a moderate impact on farmers' income.	Based on the prediction of the average monthly income of migrant workers *X*_0_(*t*) by the GM (1, 1), the income of migrant workers under the moderate impact is as follows: *X*_0_(*t*)_MES_ = *X*_0_(*t*) × (12 – *b*)/12.
Scenario 3: worse scenario (WOS)	Affected by the NCP, migrant workers cannot go out to work for half a year, which has a serious impact on farmers' income.	Based on the prediction of the average monthly income of migrant workers *X*_0_(*t*) by the GM (1, 1), the income of migrant workers under the serious impact is as follows: *X*_0_(*t*)_WOS_ = *X*_0_(*t*) × (12 – *c*)/12.

By comparing the average monthly income of migrant workers in 2020 calculated by three scenarios with the actual monthly income of migrant workers in 2019, we can analyze the impact of the COVID-19 on the income of migrant workers. Furthermore, it provides data support and suggestions for the Chinese and local governments to issue policies to return to work and protect farmers' income.

It is worth noting that due to the different policies and prevention efforts of COVID-19, the epidemic situation varies from country to country. The setting of different scenarios is the anticipation of the epidemic situation in the country. Thus, the duration of different scenarios, i.e., the range and value of parameters *a, b*, and *c*, can be changed in other countries.

## Empirical Analysis and Discussions

### Evaluation of the Impact of the COVID-19 on the Return to Work

#### Data

In order to compare the impact of the COVID-19 on the return to work of China's rural labor, we used the Baidu migration map (42) to obtain the data of population migration in and out of 366 cities in mainland China before and after the Spring Festival in 2020, as well as the matched data from the same lunar calendar period in 2019. These 366 cities include four municipalities (i.e., Beijing, Tianjin, Shanghai, and Chongqing) and another 334 prefectural-level cities and 28 county-level cities directly administered by the provinces.^2^

Since the outbreak of the COVID-19 in China started in Wuhan on January 23, 2020, we obtained the data of population immigration and emigration of 366 cities in China from January 10 (the start date of the Chunyun) to March 1, 2020. At the same time, in order to compare the impact of the COVID-19 on the return to work of China's rural labor in various cities in China, we obtained the migration data of 366 cities across China from January 21 (the start date of the Chunyun) to February 19 (the Lantern Festival) in 2019.

#### Calculation of the Impact of COVID-19 on the Return to Work of Rural Labor

First, this paper compared the impact of the COVID-19 on the return to work of China's rural labor by using the migration data on the Lantern Festival in 2019 and 2020. January 25, 2020 is the Spring Festival of the Chinese lunar calendar, February 8, 2020 is the Lantern Festival, and the Chunyun starts from January 10, 2020. Taking the sum of the migration out data before the Spring Festival from January 10 to January 24, 2020 as the denominator of Equation (1) and taking the migration in data from January 25 to February 8, 2020, the Spring Festival to the Lantern Festival, as the numerator of Equation (1), we can calculate the WRR of 366 cities as of February 8, 2020 (Chinese Lantern Festival), as shown in Table 2. It can be found that the average WRR of 366 cities at the end of February 8, 2020 was 25.25%.

**Table 2:** WRR of 366 cities in China (Feb. 8, 2020 and Feb. 19, 2019).

**No**.	**City**	**WRR-2020**	**WRR-2019**
1	Beijing	28.18%	70.63%
2	Tianjin	27.65%	71.01%
3	Shanghai	29.21%	66.19%
4	Chongqing	41.51%	100.00%
5	Shijiazhuang	19.45%	74.93%
6	Tangshan	20.20%	64.53%
7	Qinhuangdao	24.68%	76.95%
8	Handan	21.19%	70.80%
9	Xingtai	15.88%	68.86%
10	Baoding	15.12%	68.05%
…	…	…	…
363	Tiemenguan	27.84%	65.92%
364	Shuanhe	23.25%	69.65%
365	Kekedala	23.69%	74.01%
366	Kunyu	26.43%	56.41%
*T*-value		−65.314	
		(0.000)	
Average WRR		25.25%	82.34%

*This table shows the comparison of the work resumption rate (WRR) of 366 cities in China on February 19, 2019 and February 8, 2020 (i.e., Chinese Lantern Festival). For the WRR-2019, t_1_ is February 5, 2019 and t_2_ is February 19, 2019; ${t^{\prime }}_{1}$ is January 21, 2019 and ${t^{\prime }}_{2}$ is February 4, 2019. For the WRR-2020, t_1_ is January 25, 2020 and t_2_ is February 8, 2020; ${t^{\prime }}_{1}$ is January 10, 2020 and ${t^{\prime }}_{2}$ is January 24, 2020*.

Similarly, February 5, 2019 is the Spring Festival of the Chinese lunar calendar. February 19, 2019 is the Lantern Festival, and the Chunyun of 2019 starts from January 21, 2019. Taking the sum of the migration out data from January 21, 2019 to February 4, 2019 as the denominator of Equation (1) and taking the migration in data from February 5, 2019 to February 19, 2019 as the numerator of Equation (1), the WRR of 366 cities as of February 19, 2019 (Chinese Lantern Festival) can be obtained, as shown in the last column of Table 2. The average WRR of 366 cities as of February 19, 2019 was 82.34%. We found that the average WRR of China in 2020 was only 30.67% of that in the same period affected by COVID-19. We have also performed *T*-test for the difference of the mean value of 2019 and 2020 in order to ensure whether there is a difference between the WRR in 2019 and 2020. The result shows that the difference between the WRR in 2019 and 2020 is significant. As a large number of migrant workers cannot return to employment according to the original plan, China's large- and medium-sized enterprises have suffered a tremendous shock. Under the condition that the population mobility is restricted and the WRR is insufficient, most enterprises' existing orders are likely to be delayed. The costs of employees' wages, equipment maintenance, plant depreciation, etc. remain unchanged. Therefore, the normal operation of enterprises is under great pressure.

**Figure 1:**
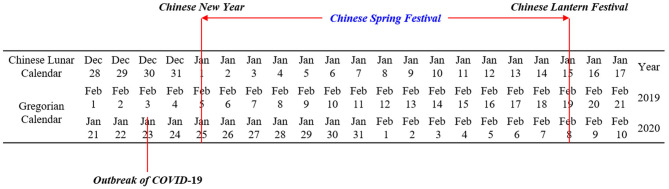
Dates of fundamental events.

**Figure 2:**
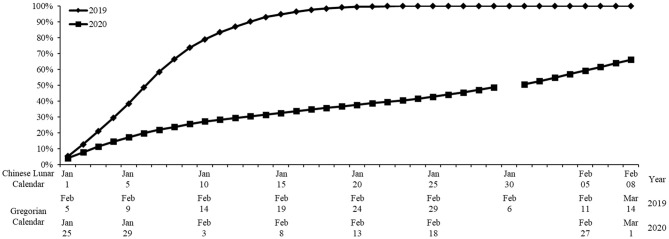
The trend of work resumption rate (WRR) in China. This figure plots the WRR in China from January 1 to February 8 based on the Chinese lunar calendar in 2019 and 2020. For WRR in 2019, *t*_1_ is February 5, 2019 and *t*_2_ are the dates from February 5 to March 14, 2019 corresponding to the horizontal axis; ${t^{\prime }}_{1}$ is January 21, 2019 and ${t^{\prime }}_{2}$ is February 5, 2019. For WRR in 2020, *t*_1_ is January 25, 2020 and *t*_2_ are the dates from January 25 to March 1, 2020 corresponding to the horizontal axis; ${t^{\prime }}_{1}$ is January 10, 2020 and ${t^{\prime }}_{2}$ is January 24, 2020. It is important to note that since 2020 is a leap year, there are only 29 days in January when we adopt the lunar calendar.

Second, with the effective control of the COVID-19, the president Xi Jinping, who hosted a meeting on February 12th, asked to conduct classified guidance to promote the work resumption in an orderly way under the premise of ensuring the epidemic prevention work (47). Figure 1 shows the correspondence between the Chinese lunar calendar and the Gregorian calendar and the dates of fundamental events. Figure 2 shows the changing trend of average WRR in China from January 1 to February 8 based on the Chinese lunar calendar in 2019 and 2020. It is obvious that the curve in 2020 after the Spring Festival is much lower than that in 2019 because of the COVID-19 pandemic. Also, the average WRR in 2020 was prone to a gradual escalation from January 25, 2020 to March 1, 2020, and it has exceeded 60%, reaching 66.17% by March 1, 2020.

**Figure 3:**
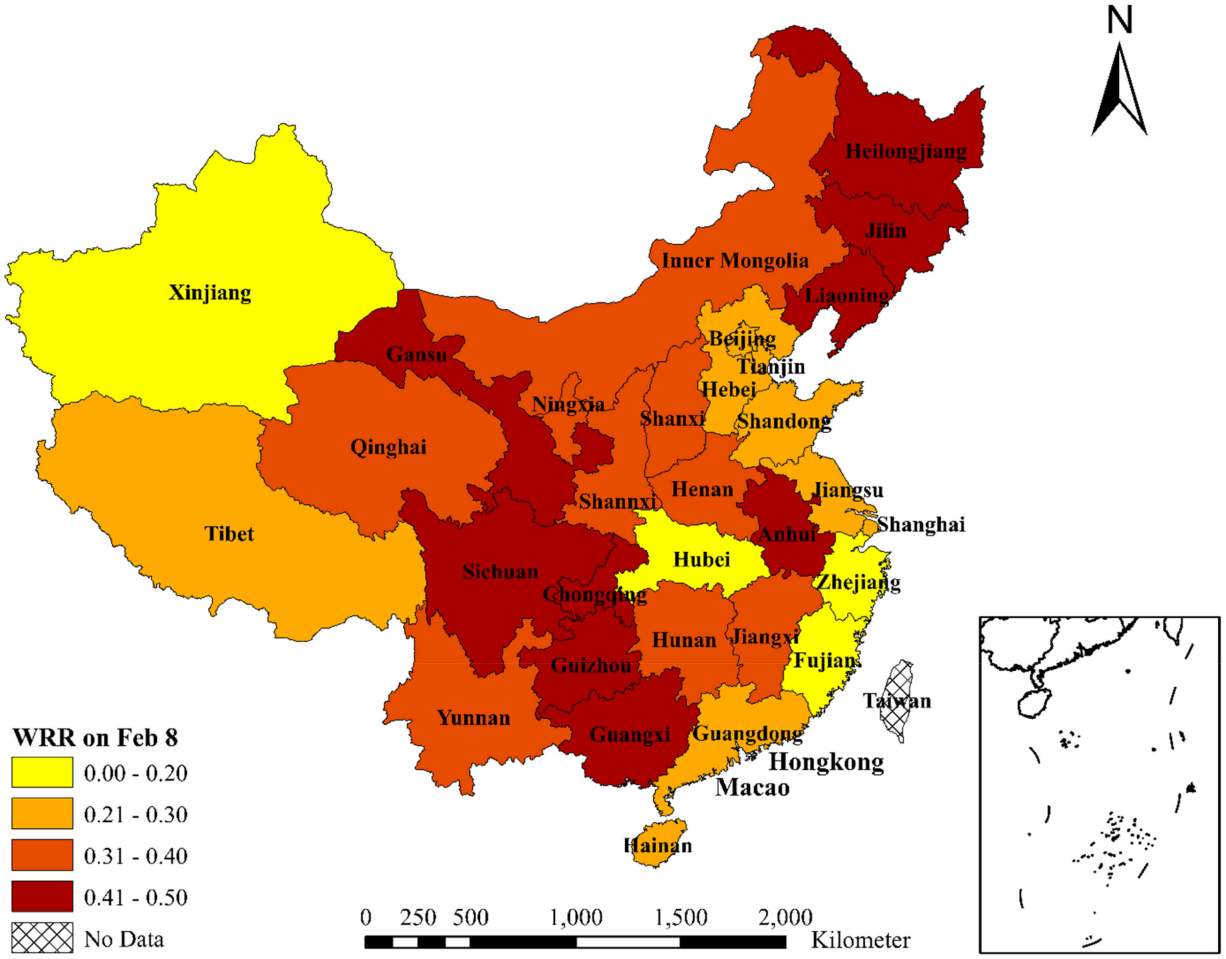
WRR of 31 Chinese provinces on February 8, 2020. This figure shows the work resumption rate (WRR) of 31 provinces in China on February 8, 2020 (i.e., Chinese Lantern Festival). For WRR on Feb 8, *t*_1_ is January 25, 2020 and *t*_2_ is February 8, 2020; ${t^{\prime }}_{1}$ is January 10, 2020 and ${t^{\prime }}_{2}$ is January 24, 2020.

**Figure 4:**
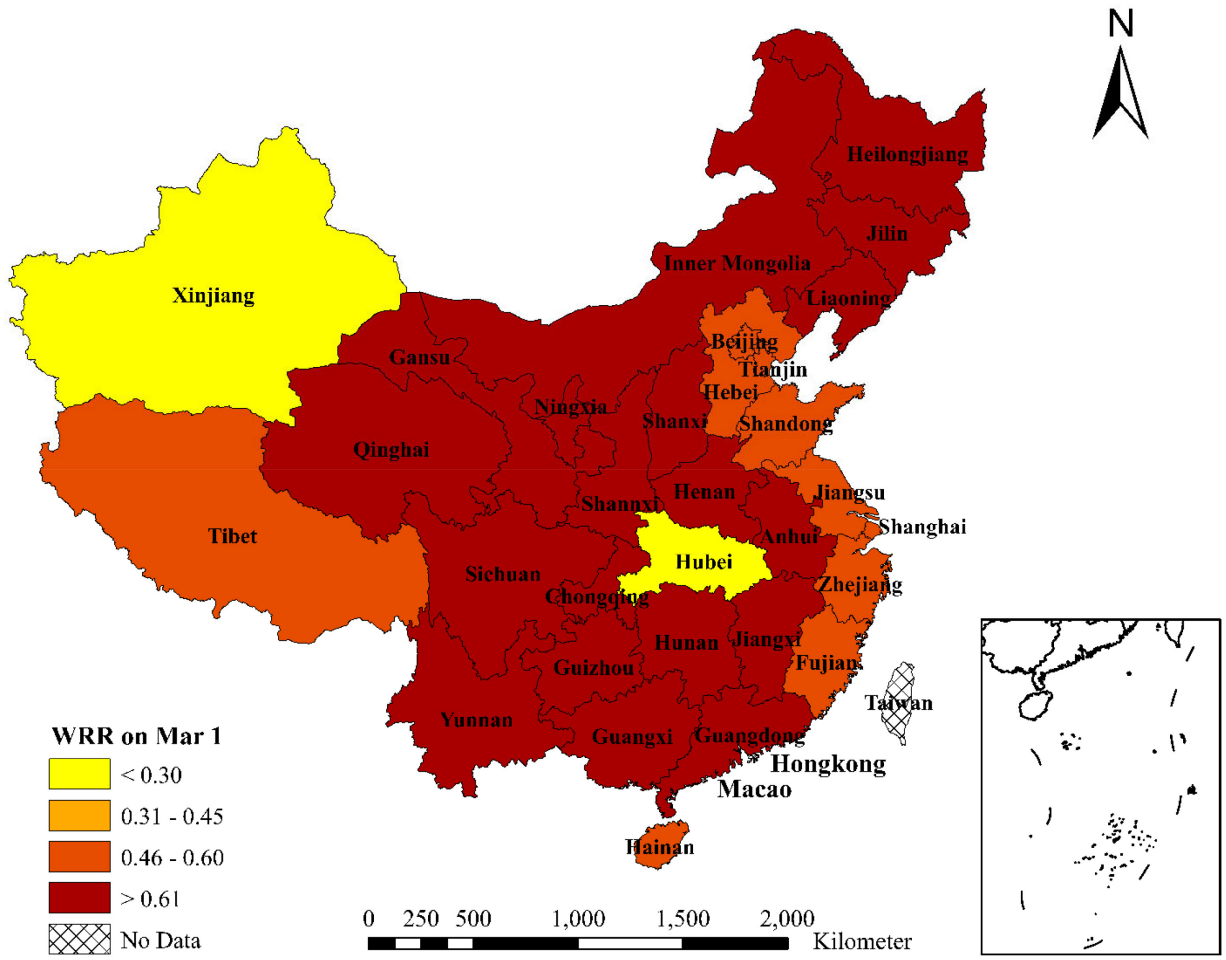
WRR of 31 Chinese provinces on March 1, 2020. This figure shows the work resumption rate (WRR) of 31 provinces in China on March 1, 2020. For WRR on March 1, *t*_1_ is January 25, 2020 and *t*_2_ is March 1, 2020; ${t^{\prime }}_{1}$ is January 10, 2020 and ${t^{\prime }}_{2}$ is January 24, 2020.

Third, the WRRs of 31 provinces in mainland China on February 8 and March 1 are shown in Table 3, and the corresponding regional distribution is shown in Figures 3, 4. Combining Table 3 with Figures 3, 4, we can find that the WRR of Zhejiang Province is the lowest in China, only 11.48%. Hubei Province, as the worst hit area of the COVID-19, has only 11.65% of the WRR. The reason is that Zhejiang province, as a major province of demanding for labor in China, mainly gets labor from Hubei, Jiangsu, Anhui, Jiangxi, and other neighboring provinces (calculated by the Baidu migration data). After the outbreak of COVID-19, the number of confirmed cases in Zhejiang has been ranked among the top five in China. Before the work resumption policy has been announced on February 10th, Zhejiang strictly implemented the policy of personnel control and enterprise shutdown, with a low WRR. Except for Hubei and Zhejiang, the WRR of Fujian and Xinjiang is also <20%, lower than the average WRR. The WRR of Sichuan, Hunan, Guizhou, and Chongqing is more than 35%, which is higher than the average WRR. The reason is that Sichuan, as a province close to Hubei and with a small number of COVID-19 confirmed case, issued policies for the full resumption of material production enterprises for epidemic prevention and control as early as January 30. Hunan, Guizhou, and Chongqing also issued relevant policies in late January or early February. Driven by these policies, workers actively returned to work. Therefore, the WRR of the above four provinces are higher.

**Table 3:** WRR of 31 provinces on February 8, 2020 and March 1, 2020.

**No**.	**Province**	**WRR on Feb 8 (%)**	**WRR on Mar 1 (%)**
1	Beijing	28.18	49.08
2	Tianjin	27.65	54.97
3	Shanghai	29.21	56.29
4	Chongqing	41.51	73.02
5	Hebei	28.51	58.31
6	Shanxi	32.37	79.89
7	Inner Mongolia	34.81	73.84
8	Liaoning	42.30	75.39
9	Jilin	48.09	77.13
10	Heilongjiang	45.60	63.19
11	Jiangsu	23.72	57.40
12	Zhejiang	11.48	49.55
13	Anhui	40.92	78.14
14	Fujian	19.31	51.71
15	Jiangxi	34.04	78.55
16	Shandong	26.36	59.29
17	Henan	34.79	70.55
18	Hubei	11.65	22.40
19	Hunan	37.96	83.48
20	Guangdong	23.46	64.69
21	Guangxi	42.91	94.07
22	Hainan	25.48	45.85
23	Sichuan	46.48	77.94
24	Guizhou	45.22	100.00
25	Yunnan	38.30	85.90
26	Tibet	21.27	52.23
27	Shaanxi	30.48	63.61
28	Gansu	44.17	88.10
29	Qinghai	38.50	76.98
30	Ningxia	32.28	63.54
31	Xinjiang	19.43	26.17

*This table shows the comparison of the work resumption rate (WRR) of 31 provinces in China on February 8, 2020 (i.e., Chinese Lantern Festival) and March 1, 2020. For the WRR on February 8, t_1_ is January 25, 2020 and t_2_ is February 8, 2020; ${t^{\prime }}_{1}$ is January 10, 2020 and ${t^{\prime }}_{2}$ is January 24, 2020. For the WRR on March 1, t_1_ is January 25, 2020 and t_2_ is March 1, 2020; ${t^{\prime }}_{1}$ is January 10, 2020 and ${t^{\prime }}_{2}$ is January 24, 2020*.

With a large number of medical workers and rescue materials rushing to Hubei, COVID-19 in China has been effectively controlled. On March 1st, most provinces began to implement the policy of work resumption. The WRR of Zhejiang Province reached to 49.55%. At the same time, because Wuhan was still the worst hit area of the COVID-19, and still took measures of city blockade, there were not many migrant workers who could successfully return to work through the “Green Channel.” The WRR of Hubei Province was 22.40%, which was higher than that on February 8, but still the lowest province in China.

In addition, we found that the WRR in Beijing, Tianjin, and Shanghai was lower than the average over the same period. The first reason is that Beijing, Tianjin, and Shanghai, as the main labor-importing cities, have a large number of returned workers, so the duration is relatively long. The second reason is that Beijing, Tianjin, and Shanghai strictly control the input personnel and implement the orderly return-to-work policy by stages and batches, resulting in the work resumption rate lower than the average in the short term. We believe that with the gradual improvement of the epidemic situation, the WRR will be significantly increased in provinces other than Hubei, which is the worst-affected area.

### Prediction of Migrant Workers' Income in 2020 Under the Impact of COVID-19

#### Prediction of Migrant Workers' Income in 2020 Under the Inertia Scenario

The average monthly income data of migrant workers from 2009 to 2019 in the second column of Table 4 was from the income statistics of migrant workers of the China National Bureau of Statistics. Substituting the data in the second column into Equations (2)–(7), the predicted income of migrant workers can be calculated by using the GM (1, 1) model, as shown in the last column of Table 4. Thus, the average error rate δ of the GM (1, 1) model could be obtained. $\delta \text{=}\frac{1}{11}\sum_{t=1}^{11}\frac{\left|{\hat{X}}_{0}(t)-X_{0}(t)\right|}{X_{0}(t)}\text{=}\frac{1}{11}\times \left(\frac{\left|207.44-207.44\right|}{207.44}\text{+}\cdots +\frac{\left|593.52-574.33\right|}{574.33}\right)\text{=}\;4.05\%$ That is to say, the accuracy of the GM (1, 1) model in predicting the average monthly income of migrant workers was 95.95%. According to the criteria of the *Prediction of Migrant Workers' Income in 2020 Under the Inertia Scenario* section above, δ = 4.05% <5%, which means that the GM (1, 1) model can be used to predict the income of migrant workers. By using Equation (7), we can get the prediction value *X*_0_ (2020) = 644.34. It can be seen that the average monthly income of migrant workers in 2020 is expected to increase by 12% compared with that in 2019 without the impact of the COVID-19, as shown in the first line of Table 5.

**Table 4:** The average monthly income of migrant workers (dollar).

**Year**	**The average monthly income of migrant workers**	**The predicted value of income**
2009	207.44	207.44
2010	249.65	288.73
2011	317.24	328.53
2012	362.77	364.93
2013	421.27	403.81
2014	466.24	441.99
2015	493.23	473.24
2016	493.05	481.75
2017	516.16	514.52
2018	562.31	569.93
2019	574.33	593.52

**Table 5:** Prediction of monthly average income of migrant workers in different scenarios.

**Scenario type**	**Predict of average monthly income of migrant workers in 2020 (dollar)**	**Income change rate compared to 2019 (%)**
Inertial scenario (INS)	644.34	12
Conservative scenario (COS)	563.80	−2
Medium scenario (MES)	456.41	−21
Worse scenario (WOS)	322.17	−44

#### Prediction of Migrant Workers' Income in 2020 Under Different Scenarios

This paper predicted the average monthly income of migrant workers in 2020 under the conservative scenario (COS). In this scenario, migrant workers cannot go out to work for 1–2 months, and the parameter belongs to the interval (1, 2). Assuming *a* = 1.5, the predicted income of migrant workers under COS can be obtained as follows: *X*_0_(2020)COS = *X*_0_(2020) × (12 – *a*)/12 = 644.34 × (12 – 1.5)/12 = 563.80. Similarly, we can calculate the average monthly income of migrant workers in 2020 under the medium scenario (MES) and worse scenario (WOS), as shown in Table 5.

It can be seen from Table 5 that the average monthly income of migrant workers will show a downward trend with the increase of the duration of COVID-19 and the strengthening of restrictions on population mobility. The greater the impact of COVID-19 on the economy and society is, the greater the decrease in migrant workers' income compared with 2019 would be. In the conservative scenario, the predicted average monthly income of migrant workers in 2020 will decrease by 2% compared with that in 2019, and the impact of COVID-19 on migrant workers' income is relatively small (see Table 5). Since the outbreak of COVID-19, the Chinese government has realized the effective administration during the epidemic through the classification of risk levels of each province and has taken quite strict prevention and control measures. It is not allowed to leave or go to the high-risk regions, but it is allowed to leave or go to the middle- or low-risk regions with the pass certificate issued by the local government. The epidemic is expected to be controlled within 1–2 months in the COS, and it can be sure that there will not be as many high-risk regions under the influence of the epidemic. Compared with the huge base of migrant workers in China, the proportion of migrant workers in high-risk regions is relatively small. In addition, the minimum living guarantee of migrant workers has been provided by the government since the epidemic. Thus, in the conservative scenario, the impact of COVID-19 on migrant workers' income is slight.

In medium scenario, migrant workers cannot go out to work for 3–4 months. Then, their average monthly income will be greatly impacted, with a decrease of about 21% compared with that in 2019. In worse scenario, migrant workers cannot go out to work for nearly half a year. Then, their average monthly income will be hugely impacted, with a decrease of about 44% compared with that in 2019. In this situation, the epidemic will have a huge impact on migrant workers' income. The government should issue emergency rescue measures to ensure the basic livelihood of farmers.

According to the National Bureau of Statistics (48), the average monthly income of migrant workers in 2020 was $590.27. In early April 2020, the pandemic has been most effectively controlled in China, and various industries have resumed work in an orderly manner; thus, it is applicable to scenario 1. Since the NBS gives the annual data, the average monthly income of migrant workers in 2020 is larger than that in April, 2020. In scenario 1, we predicted that the average monthly income of migrant workers would be $563.8, and the statistical error is (590.27 − 563.80)/590.27 = 4.49% <5%, which means that our results are robustness.

## Conclusion and Policy Implications

### Conclusion

In the context of the global outbreak of the COVID-19, it is very important to evaluate the impact of the epidemic on the return to work of rural labor and low-income groups' income such as migrant workers. It is essential to organize the labor force to return to work in an orderly manner and ensure the basic life security of low-income groups after the epidemic is stable. Based on the data of immigration and emigration of Baidu, this paper calculated the WRR of 366 Chinese cities in 2019 and 2020, respectively. According to the impact of the epidemic on migrant workers' working time, we considered three scenarios, conservative scenario (COS), medium scenario (MES), and worse scenario (WOS), and predicted the average monthly income of migrant workers in different scenarios. The results can be summarized in the following three aspects.

First, affected by the COVID-19, the average WRR was 25.25% as of February 8, 2020, which was only 30.67% of that in the same period of 2019. Since China implemented the policy of promoting to return to work, the average WRR has nearly doubled from 25.25% on February 8 to 66.17% on March 1st.

Second, the inter-provincial differences in WRR were obvious. Hubei Province, as the worst-hit area, had the lowest WRR in China. Affected by the COVID-19, the WRR in Beijing, Tianjin, Shanghai, Zhejiang, and other major labor-importing provinces were lower than the average level. As the key provinces of medical material production guarantee, Sichuan, Chongqing, Hunan, and Guizhou provinces near Hubei implemented the policy of returning to work earlier, and the WRR were relatively high.

Third, without the impact of the epidemic, the average monthly income of migrant workers in 2020 was expected to increase by 12% compared with that in 2019. With the increase of the duration of the COVID-19 and the strengthening of the government's restrictions on population mobility, the monthly average income of migrant workers will show a downward trend. Specifically, in COS that migrant workers cannot go out to work for 1–2 months, the average monthly income of migrant workers in 2020 will decrease by 2% compared with that in 2019, and the impact of the COVID-19 on migrant workers' income is relatively small. In MES that migrant workers cannot go out to work for 3–4 months, their average monthly income will be greatly impacted, with a decrease of about 21% compared with that in 2019. In WOS that migrant workers cannot go out to work for nearly half a year, their average monthly income will be hugely impacted, with a decrease of about 44% compared with that in 2019. By comparing with the data published by the National Bureau of Statistics, we found that the accuracy of our forecast is relatively high.

### Policy Implications

China has achieved a staged victory in the prevention and control of the COVID-19, and various regions have begun to resume work in an orderly manner. However, the epidemic situation is still very serious in other countries around the world such as the United States, India, Brazil, and so on. With the gradual improvement of the epidemic prevention and control in China, the experience in work resumption of China will provide an important reference for other countries in the world to formulate or adjust epidemic prevention and control measures and work resumption policies.

First, on the premise that the epidemic situation has been effectively controlled and the safety of returning to work is guaranteed, migrant workers can be organized to return to work in batches and in an orderly manner. Migrant workers should be guaranteed to return safely to work in batches and in concentration under measures such as gradually restoring road transport and opening a special railway line for them. As for the difficulties of returning to work caused by information asymmetry, local governments can use the Internet, big data, and other means to accurately connect the health status, labor skills, and the direction of intention of migrant workers with the employment demands of enterprises. The mutual recognition mechanism of health inspection for migrant workers in the inflow and outflow areas should also be adopted. We must ensure that migrant workers are allowed to go “from home to car, from car to their factory” and then to achieve a by-batch and orderly resumption.

Second, the government should strengthen employment guidance and psychological counseling for migrant workers in medium and worse scenario of the epidemic and high-risk areas, and the migrant workers should also be encouraged to find jobs nearby and locally. The local government should organize farmers to participate in online skills training and study while they are at home in order to improve their abilities. It should also be considered to increase financial investment to support migrant workers who return to their hometown to start their own businesses. Local government should also organize the spring plowing for migrant workers who are temporarily unable to return to work and make full use of public welfare position to provide migrant workers with more local employment opportunities.

Third, the central government should step up efforts to provide assistance and social security for the livelihood of low-income people such as migrant workers. The specific measures are to pay attention to the basic living security of vulnerable groups, including the families of migrant workers, and to include qualified people into the scope of social assistance. The “gradual withdrawal of subsistence allowances” mechanism should be adopted to encourage capable people to work actively and start their own businesses. For families endangering their basic survival, emergency assistance can be provided through living materials assistance, interest-free or low-interest loans, and so on to support them through their difficulties. In addition, the government should increase the subsidies for medical expenses of migrant workers' families and other difficult families and reduce the risk of poverty caused by illness in low-income families during the epidemic.

## Data Availability Statement

Publicly available datasets were analyzed in this study. This data can be found here: http://qianxi.baidu.com/.

## Author Contributions

JL and BS wrote this paper. NC, BW, and FO reviewed and improved this article. JL, BC, and BS discussed and analyzed the empirical results. All authors have read and agreed to the published version of this manuscript.

## Conflict of Interest

BC was employed by the State Grid Fujian Electric Power Co. Ltd., Fujian, China. The remaining authors declare that the research was conducted in the absence of any commercial or financial relationships that could be construed as a potential conflict of interest.

## Footnotes

^1^The Ministry of Transport of the People's Republic of China announces the specific time of the Chunyun every year. Generally, the Chunyun lasts for 40 days. The Chunyun in 2019 starts from January 21 to March 1, and the Chunyun in 2020 starts from January 10 to February 18 (http://www.mot.gov.cn/difangxinwen/xxlb_fabu/fbpd_hubei/201901/t20190118_3158463.html; http://www.mot.gov.cn/difangxinwen/xxlb_fabu/fbpd_guangdong/201912/t20191227_3314427.html).

^2^See https://en.wikipedia.org/wiki/Administrative_divisions_of_China, for more information on administrative divisions of the Republic of China.
